# Assessment of the duration of the pubertal growth spurt in patients with skeletal open bite: A cross-sectional study

**DOI:** 10.1007/s00056-020-00262-2

**Published:** 2020-12-11

**Authors:** Agnieszka Szemraj-Folmer, Anna Wojtaszek-Słomińska, Bogna Racka-Pilszak, Małgorzata Kuc-Michalska

**Affiliations:** 1 Department of Orthodontics, Faculty of Medicine, Medical University of Gdansk, Al.Zwyciestwa 42c, 80-210 Gdansk, Poland; 2 Private Orthodontic and Dental Clinic, ul.Pawliczka 10/1, 41-800 Zabrze, Poland

**Keywords:** Puberty, Age of onset, Cervical vertebrae, Cephalometry, Orthodontic treatment, Pubertät, Alter bei Beginn, Halswirbel, Kephalometrie, Kieferorthopädische Behandlung

## Abstract

**Purposes:**

The objectives were to assess the skeletal age of patients with skeletal open bite and to estimate and compare the mean chronological age related to CS3 and CS4 (cervical stage, CS) and the duration of the pubertal growth spurt (PGS).

**Methods:**

Orthodontic records of 145 patients were analyzed in this retrospective cross-sectional study. The study group comprised 104 patients with skeletal open bite (angle between cranial base plane line [NS] and mandible base plane line [ML] > 39° according to Steiner), whereas the control group comprised 41 patients with normal anteroposterior and vertical measurements. Skeletal age was assessed using the 6‑stage CVM (cervical vertebral maturation) method according to Baccetti. Quantitative variables were characterized by means of the arithmetic mean and standard deviation. The PGS duration was calculated as the difference between the means of the chronological ages related to CS3 and CS4.

**Results:**

In the study group, the arithmetic means related to CS3 and CS4 were 11.12 and 13.54 years, respectively; the duration of the PGS was 2.42 years. In the control group, the arithmetic means related to CS3 and CS4 were 10.71 and 11.82 years, respectively; the duration of the PGS was 1.11 years.

**Conclusions:**

In patients with skeletal open bite, the duration of PGS is longer but it occurs at a similar chronological age compared to patients with normal anteroposterior and vertical measurements. The PGS in males begins later than in females. Knowledge on the longer growth spurt in patients with skeletal open bite compared to patients with normal anteroposterior and vertical relationships can be useful in the selection of an appropriate therapeutic method and also provides information about the possibility of a longer and thus more effective orthopedic approach directed at positive change in the vertical growth pattern during intense bone remodeling.

## Abstract (de)

**Zielsetzungen:**

Ziele waren die Einschätzung des Knochenalters bei Patienten mit skelettal offenem Biss sowie die Einschätzung und der Vergleich des mittleren chronologischen Alters in Bezug auf CS3 („cervical stage“) und CS4 sowie die Dauer des pubertären Wachstumsschubs („pubertal growth spurt“, PGS).

**Methoden:**

In dieser retrospektiven Querschnittsstudie wurden die kieferorthopädischen Behandlungsakten von 145 Patienten analysiert. Die Studiengruppe umfasste 104 Patienten mit skelettal offenem Biss (Winkel zwischen Schädel- [NS] und Unterkieferbasisebene [ML] > 39° nach Steiner), die Kontrollgruppe umfasste 41 Patienten mit normalen anteroposterioren und vertikalen Messungen. Das Knochenalter wurde mit der 6‑stufigen CVM(„cervical vertebral maturation“)-Methode nach Baccetti bestimmt. Quantitative Variablen wurden anhand des arithmetischen Mittels und der Standardabweichung charakterisiert. Die PGS-Dauer wurde als Differenz zwischen den Mittelwerten der chronologischen Altersangaben in Bezug auf CS3 und CS4 berechnet.

**Ergebnisse:**

In der Studiengruppe betrugen die auf CS3 und CS4 bezogenen arithmetischen Mittel 11,12 bzw. 13,54 Jahre, die Dauer der PGS betrug 2,42 Jahre. In der Kontrollgruppe lagen die arithmetischen Mittel für CS3 und CS4 bei 10,71 bzw. 11,82 Jahren und die Dauer des PGS bei 1,11 Jahren.

**Schlussfolgerungen:**

Bei Patienten mit skelettal offenem Biss ist die Dauer des PGS länger, aber im Vergleich zu Patienten mit normalen anteroposterioren und vertikalen Messungen tritt es in einem ähnlichen chronologischen Alter auf. Der PGS beginnt bei Jungen später als bei Mädchen. Kenntnisse über den längeren Wachstumsschub bei Patienten mit skelettal offenem Biss im Vergleich zu Patienten mit normalem anteroposteriorem und vertikalem Verhältnis können bei der Auswahl einer geeigneten therapeutischen Methode hilfreich sein und sie vermitteln auch Informationen über die Möglichkeit eines längeren und damit effektiveren orthopädischen Ansatzes, der auf eine positive Veränderung des vertikalen Wachstumsmusters während eines intensiven Knochenumbaus abzielt.

## Introduction

In orthodontic treatment of growing patients, successful outcome depends on the proper selection of the treatment method and outcome of functional treatment is related to the optimal use of the craniofacial growth potential, i.e., the timing for orthopedic treatment is critical [1–3]. Chronological age can be one of the indicators of the maturation stage [4, 5]. However, many studies report a low correlation between chronological age and the pubertal growth spurt [4, 6, 7]. Biological age and its component skeletal age is more important [8].

The cervical vertebral maturation (CVM) method is used in orthodontics to determine the skeletal age. It is based on the assessment of cervical vertebral maturation using lateral cephalometric radiographs. This method was originally described by Lamparski in his master’s degree thesis in 1972 [9]. A modified and improved version of the method was proposed by Baccetti et al. in 2005 [2]. The use of the method allows reduction of the radiation exposure dose to the patient because there is no need to take additional hand–wrist radiographs [2, 10, 11]. Furthermore, cephalometric radiographs are routinely used in the diagnosis of patients with malocclusion [12, 13].

**Fig. 1:**
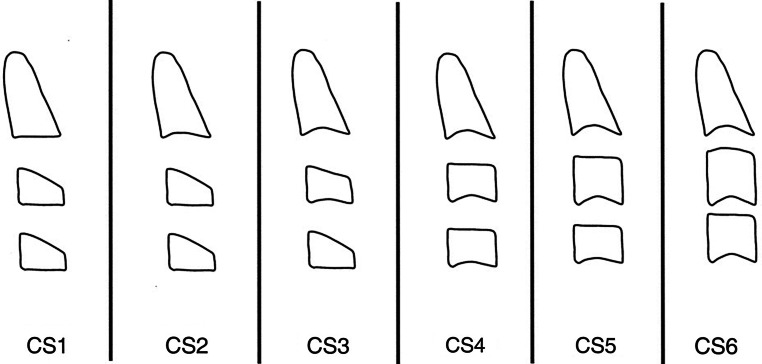
Cervical vertebral maturation (CVM) method. Schematic representation of the stages. *CS* Cervical stage. (Reprinted from [2], with permission from Elsevier) CVM(„cervical vertebral maturation“)-Methode. Schematische Darstellung der Stadien. *CS* „Cervical stage“. (Nachdruck aus [2], mit freundl. Genehmigung von Elsevier)

The CVM method is comprised of six maturational stages, i.e., from cervical stage 1 (CS1) to cervical stage 6 (CS6) [2]. The morphology of the bodies of the second, third, and fourth cervical vertebrae are analyzed (Fig. 1). They are visualized even in the presence of the radiation protective collar [14]. Vertebral shapes and the presence of concavity at the inferior border are the two most important elements in the assessment using the CVM method. Studies demonstrated the occurrence of the peak in mandibular length between CS3 and CS4 [2]. In the 2018 article *The cervical vertebral maturation method: a user’s guide*, the 20-year experience with this method is discussed [15].

The development of the skull is observed for at least two decades after birth. It is an irregular process with apposition and resorption of bone tissue. Primary and secondary maxillary and mandibular growth is observed. Primary growth is noted when the increase in dimensions is related to bone growth. However, secondary growth occurs due to the growth of other structures. The increase in the size of the facial skeleton is observed in three dimensions. The most intensive and longest increase is noted in the vertical plane. As a result, treatment of vertical malocclusion, including open bite, is difficult and therefore can last for years [16].

Skeletal open bite is mostly an extensive gnathic defect [17, 18]. There is little data in the literature on the pubertal growth spurt in patients with vertical discrepancies [19]. This knowledge would be useful in determining the proper time to start and finish active and retention treatment.

The objectives of the study was to assess the skeletal age in patients with skeletal open bite and to estimate and compare the mean chronological age related to CS3 and CS4 and the duration of the pubertal growth spurt.

## Materials and methods

This was a retrospective cross-sectional study. Patients were selected from the database of the Department of Orthodontics, Medical University of Gdańsk and Private Orthodontic and Dental Clinic in Zabrze, Poland, from 2008–2018. In order to collect patients for control and study groups, all the records from the two centers were analyzed. Common inclusion criteria for the study and control groups included CS3 or CS4 stage according to the Baccetti CVM method which is comprised of six maturation stages, age (7–18 years), good quality medical records, no previous orthodontic treatment, and no genetic syndromes. The main criterion for selecting the study group was skeletal open bite (characterized by NS/ML angle >39° according to Steiner analysis). Medical records of 1550 patients were analyzed in order to collect the study group. Of the 421 cephalometric radiographs obtained in the first stage, 133 patients were excluded due to incomplete orthodontic medical records: 99 patients due to inadequate CVM stage and 85 with a smaller NS/ML angle. The main criterion for selecting the control group was normal anteroposterior and vertical measurements. Medical records of 320 patients were analyzed to collect the control group. In the first stage, 102 patients were selected, whereby 44 patients were excluded due to skeletal malformation and 17 patients due to incomplete medical records.

The sample consisted of 145 patients. i.e., the study group comprised 104 patients with skeletal open bite and the control group was composed of 41 patients with normal anteroposterior and vertical measurements. In the groups, none of the patients had two cephalometric radiographs, i.e., one in CS3 and the other in CS4. The groups were comprised of different patients.

Imaging was performed with cephalometric and panoramic equipment (Gendex™ OrthOralix 9200, Gendex, KaVo Dental, Brea, CA, USA). Device settings were set at 8 mA, 68–72 kV and 1 s. The analysis of each radiograph was performed twice by two observers with a 1-month interval. The 6‑stage CVM method according to Baccetti was used to assess the skeletal age. Any disagreement between observers was resolved by discussion. The intra- and interobserver agreement was assessed with the kappa coefficient. The kappa values gave a reliability of 0.92 (inter-) and 0.94 (intra-) for skeletal stage maturity.

Ethical approval was obtained from the Independent Bioethical Committee at the Medical University of Gdańsk prior to data collection (NKBBN/43/2017).

### Statistical analysis

All statistical calculations were performed using Statistica software, v.13 (TIBCO® Software, 2017, TIBCO Software Inc., Palo Alto, CA, USA) and the Microsoft Excel 2010 (Microsoft, Redmond, WA, USA) spreadsheet. Quantitative variables were characterized by means of the arithmetic mean and standard deviation. The duration of the pubertal growth spurt was calculated as the difference between the means of the chronological age related to CS3 and CS4. The significance of the differences between the two groups was calculated using Student’s t test and Mann–Whitney U test. The significance level was set at *p* < 0.05.

Analysis of the power calculation for significance level of α = 0.05 confirmed that the obtained power values ≥80% were acceptable and the number of patients in each group was sufficient to demonstrate the reliability of the obtained results.

## Results

The skeletal age of patients during the pubertal growth spurt was assessed using descriptive statistics (Table 1). The minimum age related to CS3 in patients from the control group was 9 years, while in the study group it was 7.42 years. The minimum age in the female group with normal anteroposterior and vertical measurements was 1 year and 1 month lower compared to males (9 and 10.08 years, respectively). In the study group, the minimum age related to CS3 in females was 2 years lower than in males (7.42 and 9.42 years, respectively). The maximum age in the control group related to CS4 was 14.92 years in males and 11.92 years in females. However, in patients from the study group the values were 17.42 and 17.25 years, respectively.

**Table 1:** Characteristics of the chronological age in the control and study groups Eigenschaften des chronologischen Alters in der Kontroll- und in der Studiengruppe

	No. of records	Minimum	Maximum	Mean age (years)	SD
*Control group MEN* *+* *WOMEN*	41	9.00	14.92	11.09	1.25
*Control group WOMEN*	29	9.00	11.92	10.77	0.96
*Control group MEN*	12	10.08	14.92	11.89	1.54
*Study group MEN* *+* *WOMEN*	104	7.42	17.42	12.27	2.12
*Study group WOMEN*	69	7.42	17.25	11.94	2.09
*Study group MEN*	35	9.42	17.42	12.92	2.05

*CS* cervical stage, *SD* standard deviation

Tables 2 and 3 show the comparison of the arithmetic means of the chronological age related to CS3 and CS4 and the difference between these maturation stages that shows the duration of the pubertal growth spurt. In the study group, the arithmetic mean related to CS3 was 11.14 years, whereas in the case of CS4 it was 13.54 years. These subgroups were statistically different (*p* = 0.001). The duration of the pubertal growth spurt was 2.40 years. In the control group, the arithmetic mean related to CS3 was 10.71 years, whereas in the case of CS4 it was 11.82 years. These subgroups were also statistically different (*p* = 0.014) and the duration of the pubertal growth spurt was 1.11 years.

**Table 2:** Estimation and comparison of the mean chronological age related to CS3 and CS4 and the duration of the craniofacial pubertal growth spurt in the control group Einschätzung und Vergleich des mittleren chronologischen Alters in Bezug auf CS3 und CS4 und Dauer des kraniofazialen pubertären Wachstumsschubs in der Kontrollgruppe

	CVM stage	No. of records	Mean age (years)	SD	CVM years (3–4)	*Z*	*p*
*Control group MEN* *+* *WOMEN*	CS3	27	10.71	0.97	1.11	2.45	0.014^*^
	CS4	14	11.82	1.44			
*Control group WOMEN*	CS3	19	10.57	1.01	0.58	1.54	0.124
	CS4	10	11.15	0.76			
*Control group MEN*	CS3	8	11.07	0.81	2.45	2.64	0.008*
	CS4	4	13.52	1.37			

*CVM* cervical vertebral maturation, *CS* cervical stage, *SD* standard deviation

*statistically significant at *p* < 0.05

**Table 3:** Estimation and comparison of the mean chronological age related to CS3 and CS4 and the duration of the craniofacial pubertal growth spurt in the study group Einschätzung und Vergleich des mittleren chronologischen Alters in Bezug auf CS3 und CS4 und Dauer des kraniofazialen pubertären Wachstumsschubs in der Studiengruppe

	CVM stage	No. of records	Mean age (years)	SD	CVM years (3–4)	*t*	*Df*	*p*
*Study group MEN* *+* *WOMEN*	CS3	55	11.14	1.65	2.40	6.96	102	0.001^*^
	CS4	49	13.54	1.87				
*Study group WOMEN*	CS3	35	10.60	1.54	2.73	7.14	67	0.001^*^
	CS4	34	13.33	1.63				
*Study group MEN*	CS3	20	12.10	1.37	1.92	3.05	33	0.004^*^
	CS4	15	14.02	2.32				

*CVM* cervical vertebral maturation, *CS* cervical stage, *SD* standard deviation

Table 4 shows the comparison of all results. Means of the chronological age related to CS3 in the control and the study groups were not significantly different (*p* = 0.115) as opposed to the values at CS4, which were significantly different (*p* = 0.002).

**Table 4:** Estimation and comparison of the mean chronological age related to CS3 and CS4 Einschätzung und Vergleich des mittleren chronologischen Alters in Bezug auf CS3 und CS4

	CVM stage	No. of records	Mean age (years)	SD	*Z*	*p*
*Control group MEN* *+* *WOMEN*	CS3	27	10.71	0.97	1.57	0.115
*Study group MEN* *+* *WOMEN*	CS3	55	11.14	1.64		
*Control group MEN* *+* *WOMEN*	CS4	14	11.82	1.44	3.17	0.002^*^
*Study group MEN* *+* *WOMEN*	CS4	49	13.54	1.87		

*CVM* cervical vertebral maturation, *CS* cervical stage, *SD* standard deviation

## Discussion

There are relatively few reports in the literature regarding the duration of the pubertal growth spurt in patients with vertical discrepancies [19, 20]. Celebri et al. [19] analyzed peak timing in patients with sagittal and vertical discrepancies. Patients were divided into 12 groups, i.e., females and males separately with skeletal class I, II, and III, normal facial pattern, high angle and low angle patients. No significant differences were observed between the growth patterns. The discrepancies between studies may result from smaller group sizes. The group of high-angle patients consisted of only 35 subjects. Differences may also be due to their use of another method. To assess the skeletal age, those authors used the hand–wrist method according to Greulich and Pyle in which there is no stage correlating with the onset timing of the pubertal growth spurt. There is only the MP3cap (medial phalanges III, capping of epiphysis to diaphysis) stage that was considered the peak stage. Both studies demonstrated earlier occurrence of the peak stage in females compared to males.

In the present study, the duration of the pubertal growth spurt in patients with skeletal open bite was more than twice as long compared to patients with normal facial pattern. It can therefore be assumed that increased vertical craniofacial growth is probably associated with a longer pubertal spurt. Similar observations were reported by researchers involved in studies on skeletal class III (progenia) [21–23], in which the duration of the pubertal growth spurt was 9.72–11 months (0.8–0.9 year) in control groups (patients with normal anteroposterior and vertical measurements) and 14.52–17 months (1.19–1.4 years) in class III patients. Those authors reported that increased mandibular growth may result from longer pubertal growth spurt.

Another interesting observation from the above studies is a similar arithmetic mean of the age related to the onset of the pubertal growth spurt in the control and study groups, which means that in patients with both skeletal class I and III, the most intensive growth is observed in the similar chronological age. Our study results are in line with the above observations. Although the minimum age in patients with skeletal open bite was lower compared to patients from the control group, assessment of the arithmetic means showed that the chronological age related to CS3 was not significantly different between the two groups.

Of note, the assessment of the study results showed the discrepancy between females and males. For instance, the duration of the pubertal growth spurt in the female control group was 0.58 and 2.45 years in the male group. The reason for such differences may be related to a smaller sample size of males. Females are more likely to have orthodontic appointments probably due to the fact that they attach more importance to facial esthetics and appearance compared to males.

Determination of skeletal age in patients with malocclusion is useful in planning orthodontic treatment [24, 25]. Knowledge on the longer growth spurt in patients with skeletal open bite compared to patients with normal anteroposterior and vertical relationships can be useful when selecting an appropriate therapeutic method and also provides information about the possibility of a longer and thus more effective orthopedic approach directed at positive change in the vertical growth pattern during intense bone remodeling [26]. The initiation of orthodontic and surgical treatment in such cases should be conducted later due to the possible lack of stable treatment effect [27].

Reports in the literature on the early treatment of open bite is still controversial and covers many different therapeutic approaches [28]. Baccetti et al. [29] showed that treatment with rapid maxillary expander (RME) and vertical-pull chincup (V-PCC) therapy used in the pubertal growth spurt was more effective and resulted in better outcomes compared to prepubertal treatment. In addition, skeletal discrepancies are usually more pronounced during the pubertal growth spurt. Therefore, the ability to predict the growth potential seems to be essential in the diagnosis of patients with malocclusions and in prevention of further discrepancies [2, 30, 31].

Craniofacial growth takes place in three planes and is a long-term process. According to Behrents [32], facial development is also found to some extent into adulthood. The growth process in each plane is characterized by different acceleration and length. Changes in the vertical plane of the craniofacial skeleton in the late teens are definitely more noticeable than anteroposterior changes [16]. Studies also indicate that most skeletal changes in the vertical plane occur between adolescence and mid-adulthood [33].

The above observations were the basis for determining the criterion of age for patients namely 7–18 years. The arithmetic mean in CS4 stage for both sexes was 12.27 years. Therefore, it was significantly lower than the upper limit of the criterion. However, 5 of 104 patients turned 16 years of age. These data may be of scientific importance since they suggest that there is a group of high-angle patients whose adolescent growth lasts longer.

The study patients were characterized by a small range of NS/ML angle (40–43°). A correlation between the severity of the malocclusion and the length of the pubertal growth spurt was not found. However, this type of analysis could be performed again in the future in cross-sectional studies with a larger sample of patients.

There are many supporters and opponents of the CVM method. The opponents stress the lack of specifically defined stages, which results in poor reproducibility of the results [34–36]. In 2018, McNamara et al. [15] published *The cervical vertebral maturation method: a user’s guide*, which showed that the reproducibility of the CVM method improved with the clinician’s experience. In addition, those authors clearly demonstrated that changes in morphology of the cervical vertebrae are gradual and continuous—“A person does not go to bed in the evening at CS 2 and wake up the next morning at CS 3”. In their paper, each stage was described very precisely and included cervical vertebrae radiographs (CS1–CS6).

Over the past decades, accelerated puberty has been observed [37–39]. Many factors influence the child’s growth rate, including genetic determinants, nutrition, climate, hormonal disorders, and environmental influences [40]. Thus, chronological age is not a good indicator to assess growth stage.

Of note, the results of the present study are obtained from cross-sectional data and do not reflect true changes that occur in the maturation process. However, the total number of subjects in the study and a significant difference in the duration of the pubertal growth spurt between the control and the study groups confirm the statistical significance of the results. Currently, future studies that could show the real duration of the growth spurt do not seem possible due to radiation protection. Exposure of patients to several radiation doses in the developmental period would be contrary to the goal of minimizing radiation dose.

## Conclusions

- Pubertal growth spurt in patients with skeletal open bite is longer than in patients with normal anteroposterior and vertical measurements.
- The onset of the pubertal growth spurt in patients with normal anteroposterior and vertical measurements and skeletal open bite is at similar chronological age.
- Pubertal growth spurt in males begins later than in females.
- Knowledge on the longer growth spurt in patients with skeletal open bite compared to patients with normal anteroposterior and vertical relationships can be useful in the selection of an appropriate therapeutic method and also provides information about the possibility of longer and thus more effective orthopedic approach directed at positive change in the vertical growth pattern during intense bone remodeling.
